# Author Correction: The AP-1 transcription factor Fosl-2 drives cardiac fibrosis and arrhythmias under immunofibrotic conditions

**DOI:** 10.1038/s42003-023-04600-z

**Published:** 2023-02-23

**Authors:** Mara Stellato, Matthias Dewenter, Michal Rudnik, Amela Hukara, Çagla Özsoy, Florian Renoux, Elena Pachera, Felix Gantenbein, Petra Seebeck, Siim Uhtjaerv, Elena Osto, Daniel Razansky, Karin Klingel, Joerg Henes, Oliver Distler, Przemysław Błyszczuk, Gabriela Kania

**Affiliations:** 1 Center of Experimental Rheumatology, Department of Rheumatology, University Hospital Zurich, University of Zurich, Zurich, Switzerland; 2 Institute of Experimental Cardiology, University Hospital, Heidelberg, Germany; 3 Institute for Biomedical Engineering and Institute of Pharmacology and Toxicology, Faculty of Medicine, University of Zurich, Zurich, Switzerland; 4 Institute for Biomedical Engineering, Department of Information Technology and Electrical Engineering, ETH Zurich, Zurich, Switzerland; 5 Zurich integrative Rodent Physiology, University of Zurich, Zurich, Switzerland; 6 Institute of Clinical Chemistry, University Hospital Zurich, Zurich, Switzerland; 7 Cardiopathology, Institute for Pathology and Neuropathology, University Hospital Tubingen, Tubingen, Germany; 8 Internal Medicine II, Division of Rheumatology, University Hospital Tubingen, Tubingen, Germany; 9 Department of Clinical Immunology, Jagiellonian University Medical College, Krakow, Poland

**Keywords:** Rheumatic diseases, Cell biology

Correction to: *Communications Biology* 10.1038/s42003-023-04534-6, published online 09 February 2023.

In this article, the affiliation details for Matthias Dewenter were incorrectly given as ‘Institute for Biomedical Engineering and Institute of Pharmacology and Toxicology, Faculty of Medicine, University of Zurich, Zurich, Switzerland’.

In this article, the affiliation ‘Institute for Biomedical Engineering and Institute of Pharmacology and Toxicology, Faculty of Medicine, University of Zurich, Zurich, Switzerland’ for Çagla Özsoy and Daniel Razansky was missing.

The original article has been corrected.

