# Supplementary material for: The AP-1 transcription factor Fosl-2 drives cardiac fibrosis and arrhythmias under immunofibrotic conditions
Source: Commun Biol. 2023 Feb 9;6:161. doi: 10.1038/s42003-023-04534-6 (PMC9911788; doi:10.1038/s42003-023-04534-6)
Supplement: Supplementary file 2 — Supplementary Information [file 42003_2023_4534_MOESM2_ESM.pdf]

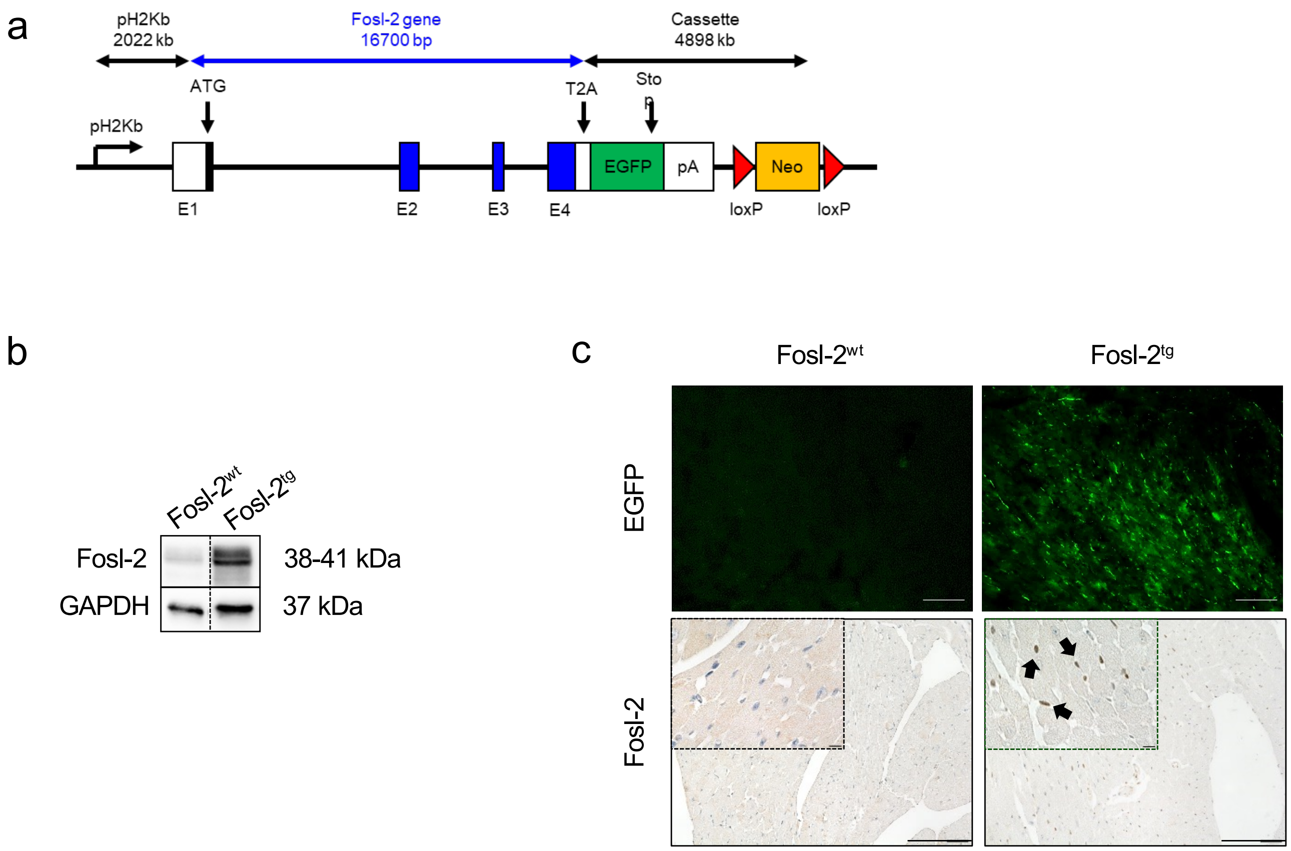

Supplementary Figure 1. Fosl-2 transgenic mouse model

(a) Schematic composition of the genetic vector used for the generation of Fosl-2^{tg} mice. The murine Fosl-2 gene, contains 4 exons (E1 to E4) and it is expressed under the control of H2Kb promotor. The EGFP coding sequence was inserted in frame with the Fosl-2 gene as a tracer, with a self-cleaving peptide sequence T2A between them to generate two separated proteins. (b) WB analysis of Fosl-2 protein content in homogenized hearts isolated from 16-22-week-old Fosl-2^{wt} and Fosl-2^{tg} mice. (c) The upper panel shows representative pictures of OCT-embedded cardiac sections from Fosl-2^{wt} and Fosl-2^{tg} where EGFP expression is visible in green. In the lower panel: paraffin-embedded myocardial sections from Fosl-2^{wt} and Fosl-2^{tg} stained for Fosl-2. Arrows indicate nuclear expression of Fosl-2. Scale bars: 100 µm, insert scale bars: 50 µm.

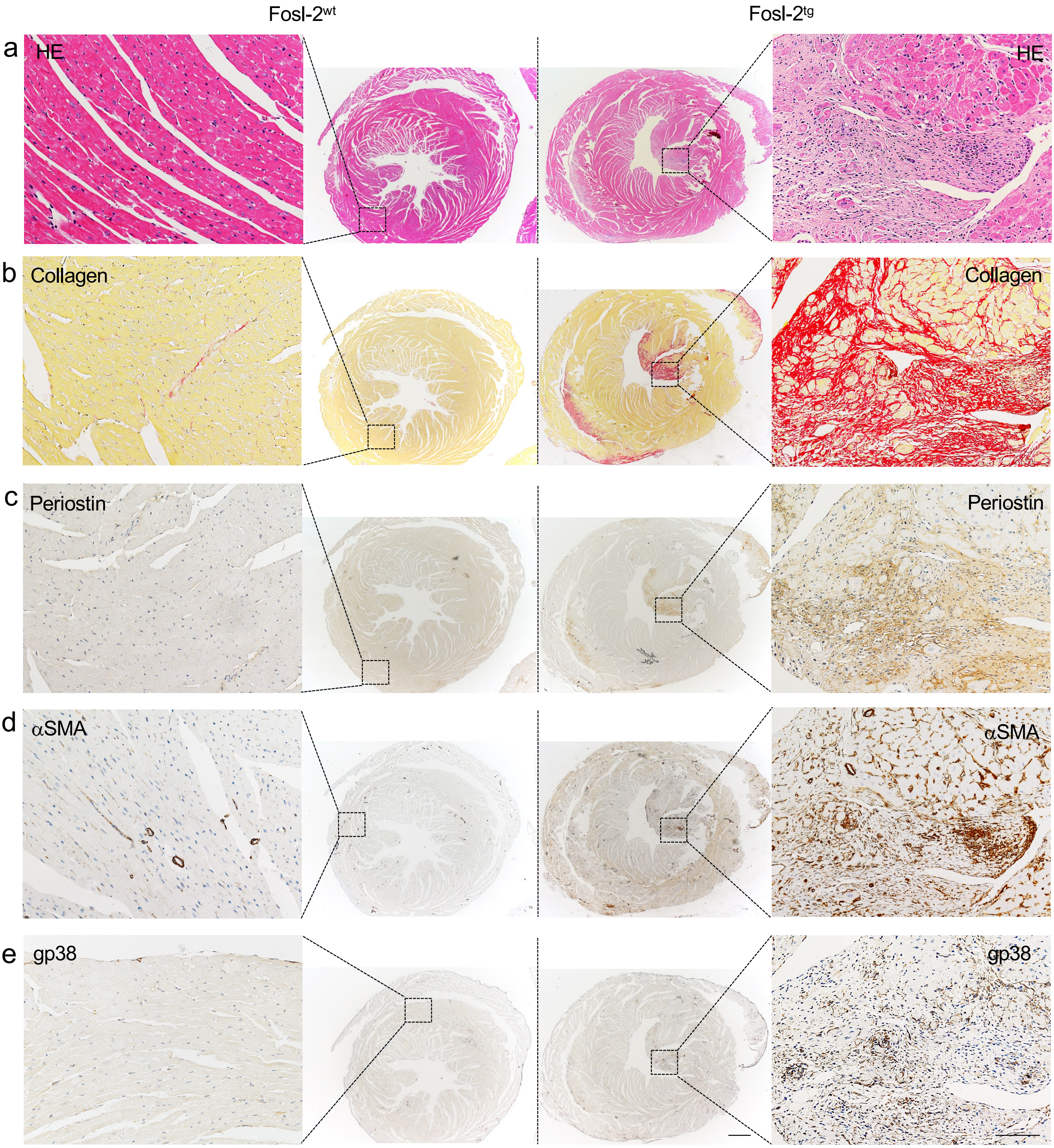

Supplementary Figure 2. Fibrotic phenotype in the heart of 16-22-week-old Fosl-2^{tg} mice

Representative pictures of HE (a), Sirius Red staining for collagen (b), IHC for periostin (c), αSMA (d) and gp38 (podoplanin) (e) of myocardial sections from 16-22-week-old mice. (Scale bar: 500 µm, insert scale bar: 100 µm.

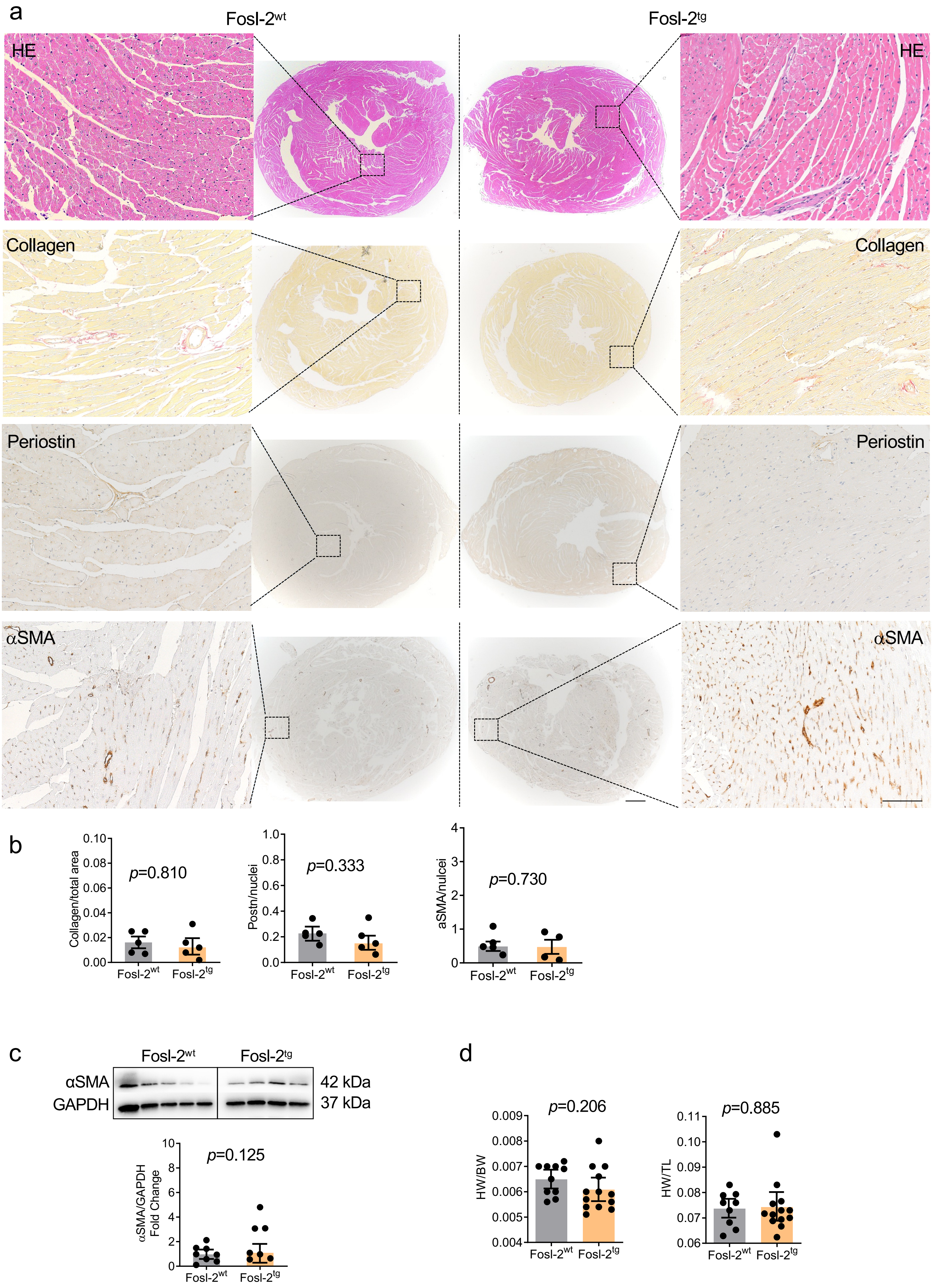

a
Fosl-2wt
Fosl-2tg
HE
Collagen
Periostin
αSMA
b
p=0.810
Collagen/total area
0.10
0.08
0.06
0.04
0.02
0.00
Fosl-2wt
Fosl-2tg
p=0.333
Postn/nuclei
1.0
0.8
0.6
0.4
0.2
0.0
p=0.730
aSMA/nulcei
4
3
2
1
0
c
αSMA
GAPDH
42 kDa
37 kDa
p=0.125
αSMA/GAPDH Fold Change
10
8
6
4
2
0
d
p=0.206
HW/BW
0.009
0.008
0.007
0.006
0.005
0.004
p=0.885
HW/TL
0.11
0.10
0.09
0.08
0.07
0.06

Supplementary Figure 3. Assessment of the phenotype in the heart of 7-week-old Fosl-2^{tg} mice

(a) Representative pictures for HE, Sirius Red staining for collagen, IHC for periostin and αSMA, and (b) quantification of Sirius Red staining, periostin (Postn) and αSMA of myocardial sections from 7-week-old mice (n=5) (*t-test,* mean ± SEM). Scale bar: 500 µm, insert scale bar: 100 µm. (c) Representative WB and densitometric analysis of αSMA protein content in homogenized hearts isolated from 7-week-old mice (n=7-8, *t-test,* mean ± SEM). (d) Heart weight (HW)/body weight (BW) and HW/tibial length (TL) ratios at week 7 (n=10-13, *t-test,* mean ± SEM).

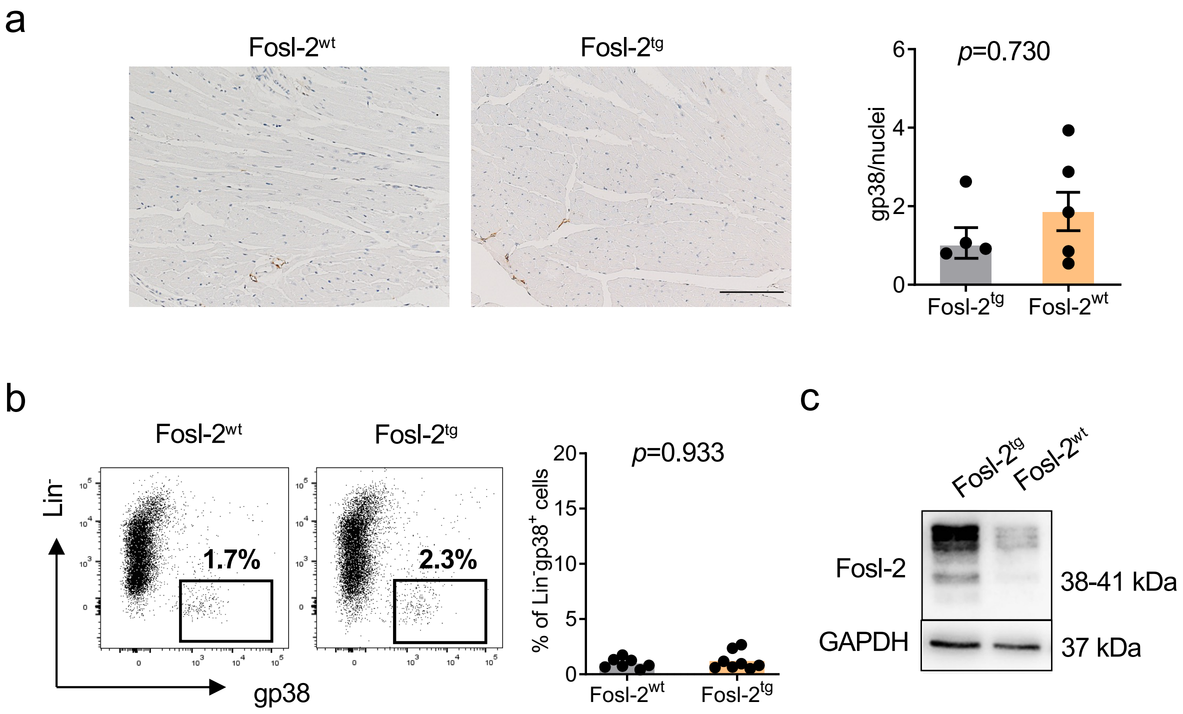

Supplementary Figure 4. Lin^-gp38^+ cell phenotyping before SSc myocardial fibrogenesis

(a) Representative pictures and corresponding quantification of myocardial sections of gp38 IHC staining from 7-week-old mice (n=4-5, *Mann-Whitney test,* mean ± SEM). Scale bar: 100 µm. (b) Representative flow cytometry pictures and corresponding quantification of Lin^-gp38^+ fibroblast frequency from the myocardium of 7-week-old mice (n=8, *Mann-Whitney test,* mean ± SEM). (c) Representative pictures of WB for Fosl-2 protein expression in lysates from cultured $Lin^-gp38^+Fosl\text{-}2^{wt}$ and $Lin^-gp38^+Fosl\text{-}2^{tg}$ fibroblasts. $Lin^-gp38^+Fosl\text{-}2^{wt}$ and $Lin^-gp38^+Fosl\text{-}2^{tg}$ fibroblasts were FACS-sorted and in vitro cultured (n=3).

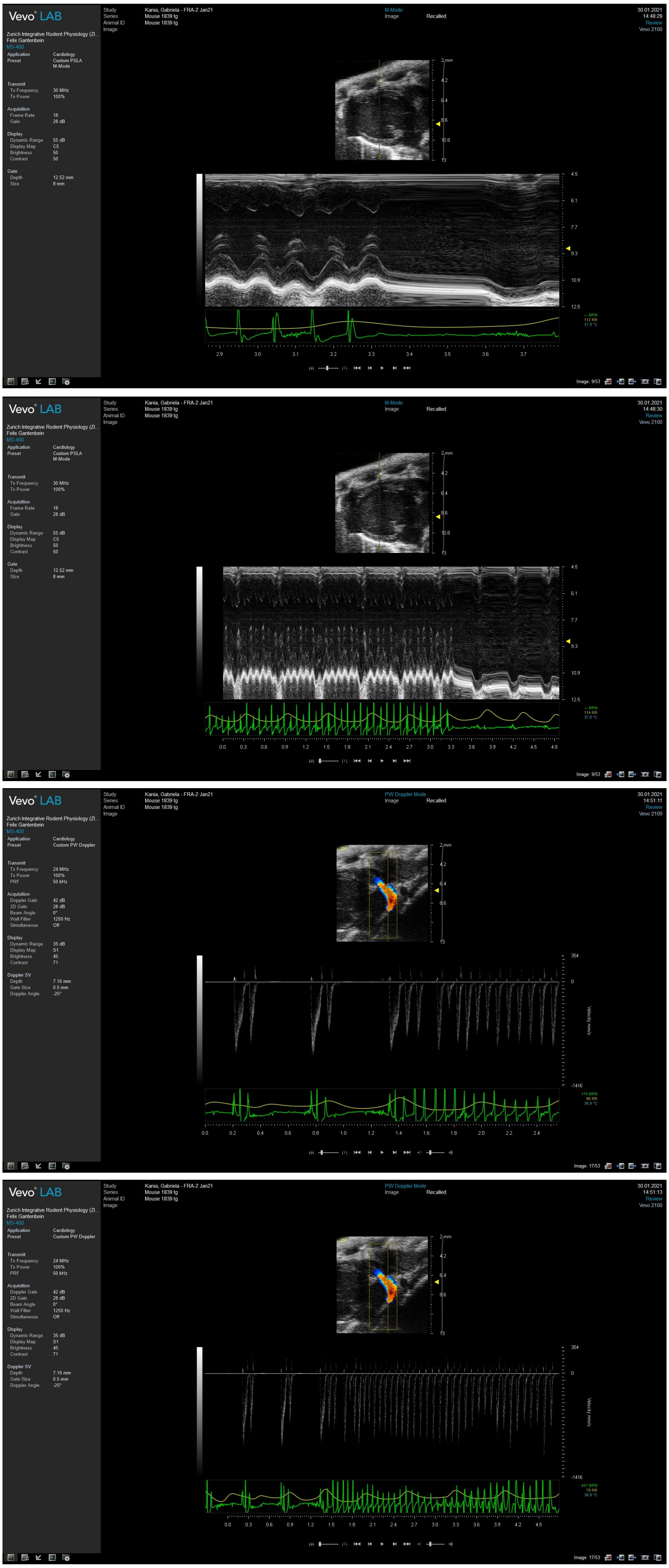

Supplementary Figure 5. Cardiac arrhythmias observed during the echocardiography in Fosl-2^{tg} mouse at week 20

Paroxysmal tachycardia observed in a Fosl-2^{tg} mouse during echocardiography under isoflurane/O2 anaesthesia. The heart rhythm during the 15-minute long exam alternated between phases of 1-3 tachycardic events of <5 seconds each (mostly preceded and followed by several heartbeat triplets) and 1-3 minute-long phases of sinus rhythm.

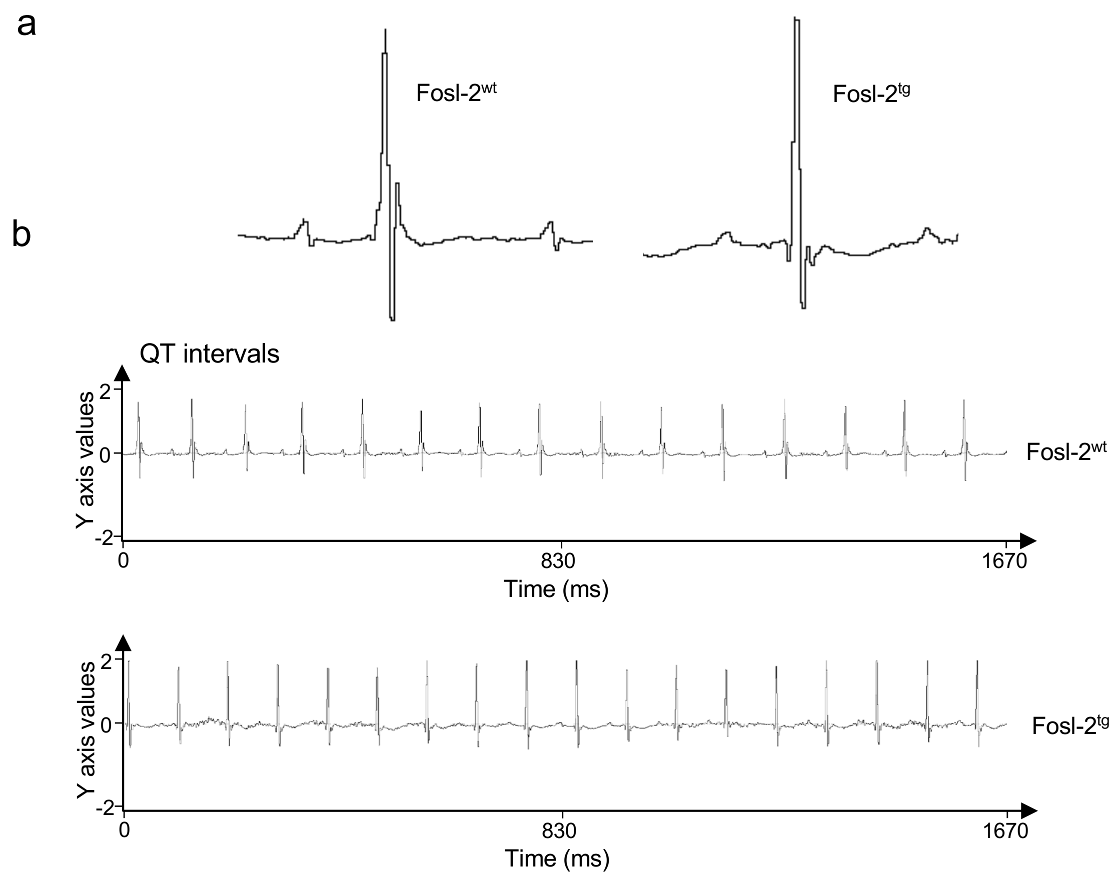

Supplementary Figure 6. ECG recordings in Fosl-2^{wt} and Fosl-2^{tg} mice

(a) Representative pictures of radiotelemetry ECG recorded in Fosl-2^{wt} and Fosl-2^{tg} mice at week 20-22. (b) Representative radiotelemetry ECG pictures of QT interval in Fosl-2^{wt} and Fosl-2^{tg} mice at week 20-22.

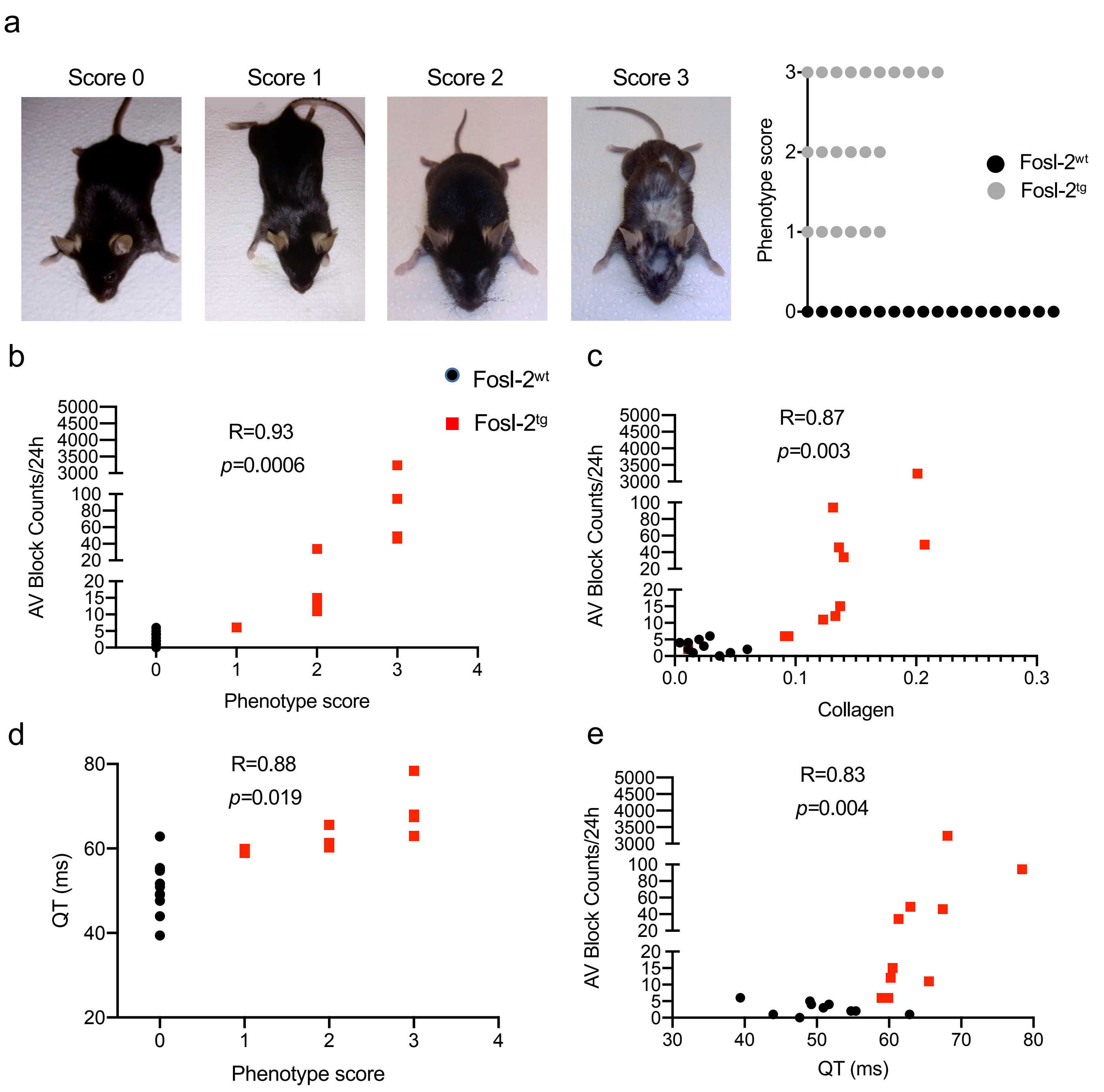

Supplementary Figure 7. Phenotype severity in Fosl-2^{tg} mice

(a) Assessment of the gross phenotype severity in Fosl-2^{tg} mouse cohort analysed by assignment of phenotype disease scores (no lesions: score 0, only dermatitis: score 1, severe dermatitis around eyes or blindness: score 2, dermatitis around eyes and diffused skin lesions on the body: score 3; n=22). Fosl-2^{wt} phenotype was set as 0 (n=18). (b-c) Spearman correlation between AV block counts and phenotype score (b), and between AV block counts and collagen expression quantified by Sirius Red staining (c) (n=10). AV block counts were assessed by radiotelemetry. (d-e) Spearman correlation between QT intervals and phenotype score (d), and between QT intervals and AV block counts (e) (n=10). QT intervals and AV block counts were assessed by radiotelemetry. AV block: atrio-ventricular block.

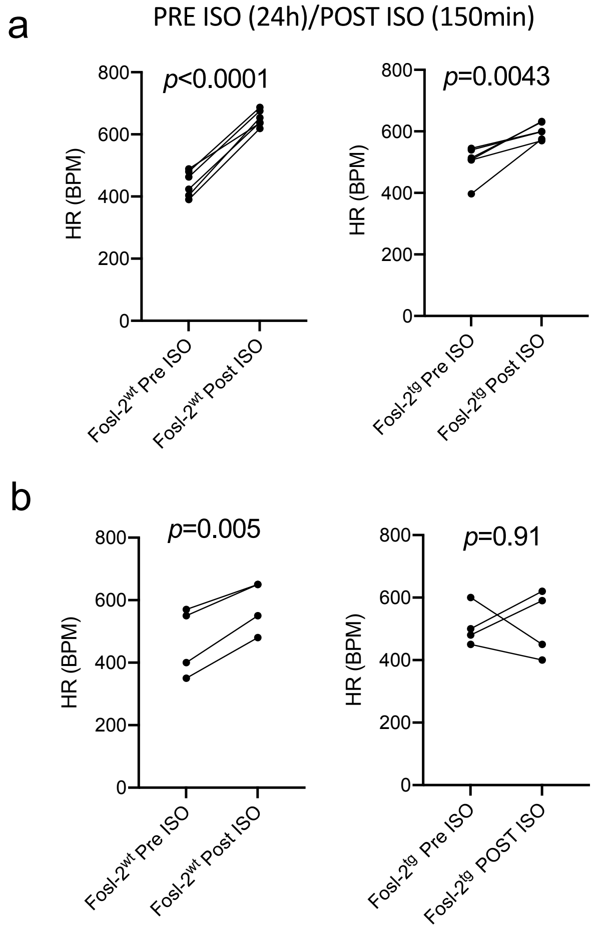

Supplementary Figure 8. Alteration in heart rate in Fosl-2^{tg} mice under the stress condition

(a) Analyses of HR (BPM) changes before isoproterenol (ISO) (PRE ISO, 24h) and after ISO (POST ISO, 150min) stimulation in Fosl-2^{wt} or Fosl-2^{tg} mice (n=6, *paired t-test*). (b) Analyses of HR (BPM) changes before ISO (PRE ISO) and after ISO (POST ISO) stimulation of Fosl-2^{wt} and Fosl-2^{tg} hearts in *ex-vivo* Langendorff reperfusion system (n=4, *t-test*). BPM: beats per minute.

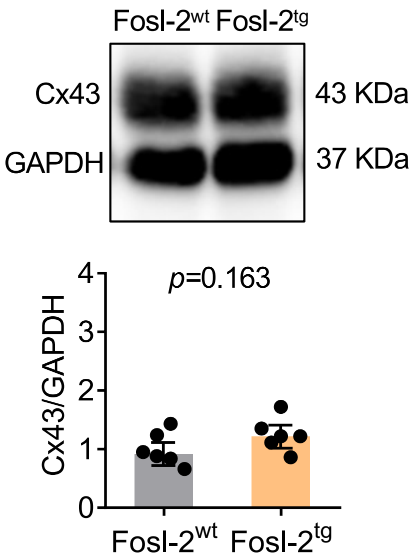

Supplementary Figure 9. Connexin (Cx) 43 in the heart of Fosl-2^{wt} and Fosl-2^{tg} mice Representative WB blot and densitometry analysis of WB analysis for the expression of total Cx43 protein content in lysates from homogenized hearts of 16-22-week-old Fosl-2^{wt} and Fosl-2^{tg} mice (n=6, *t-test,* mean ± SEM).

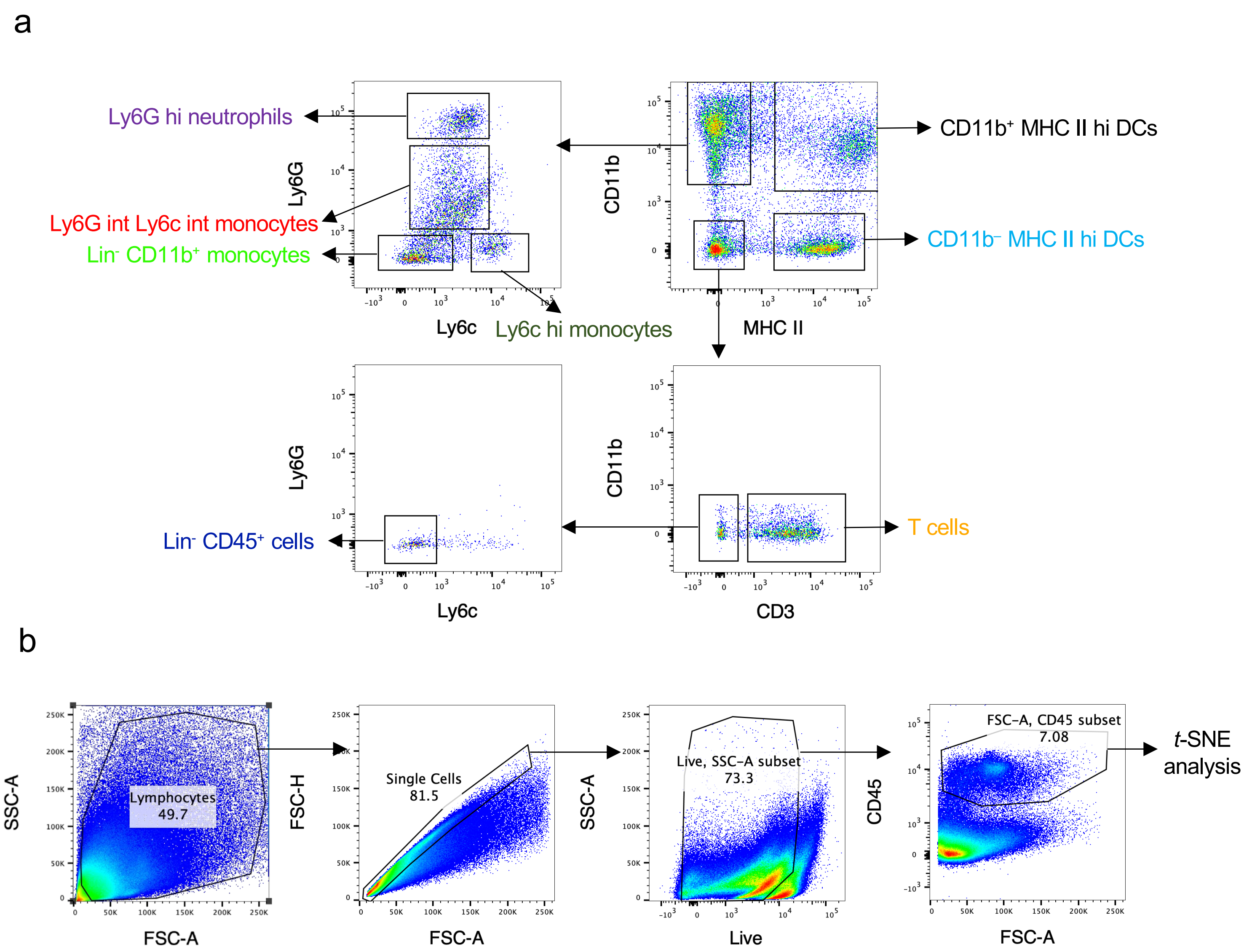

Supplementary Figure 10. Targeting inflammation in the mouse heart

(a) Gating strategy in flow cytometry analysis of immune cells in representative mouse heart for the identification of Ly6G high (hi) neutrophils, Ly6G intermediate (int) monocytes, Ly6c int monocytes, Lin$^-$CD11b$^+$ monocytes, CD11b$^+$MHCII hi dendritic cells (DCs), CD11b$^-$MHCII hi DCs, Lin$^-$CD45$^+$ cells and T cells. (b) Gating strategy for investigating cardiac immune cell populations for *t*-SNE analysis.

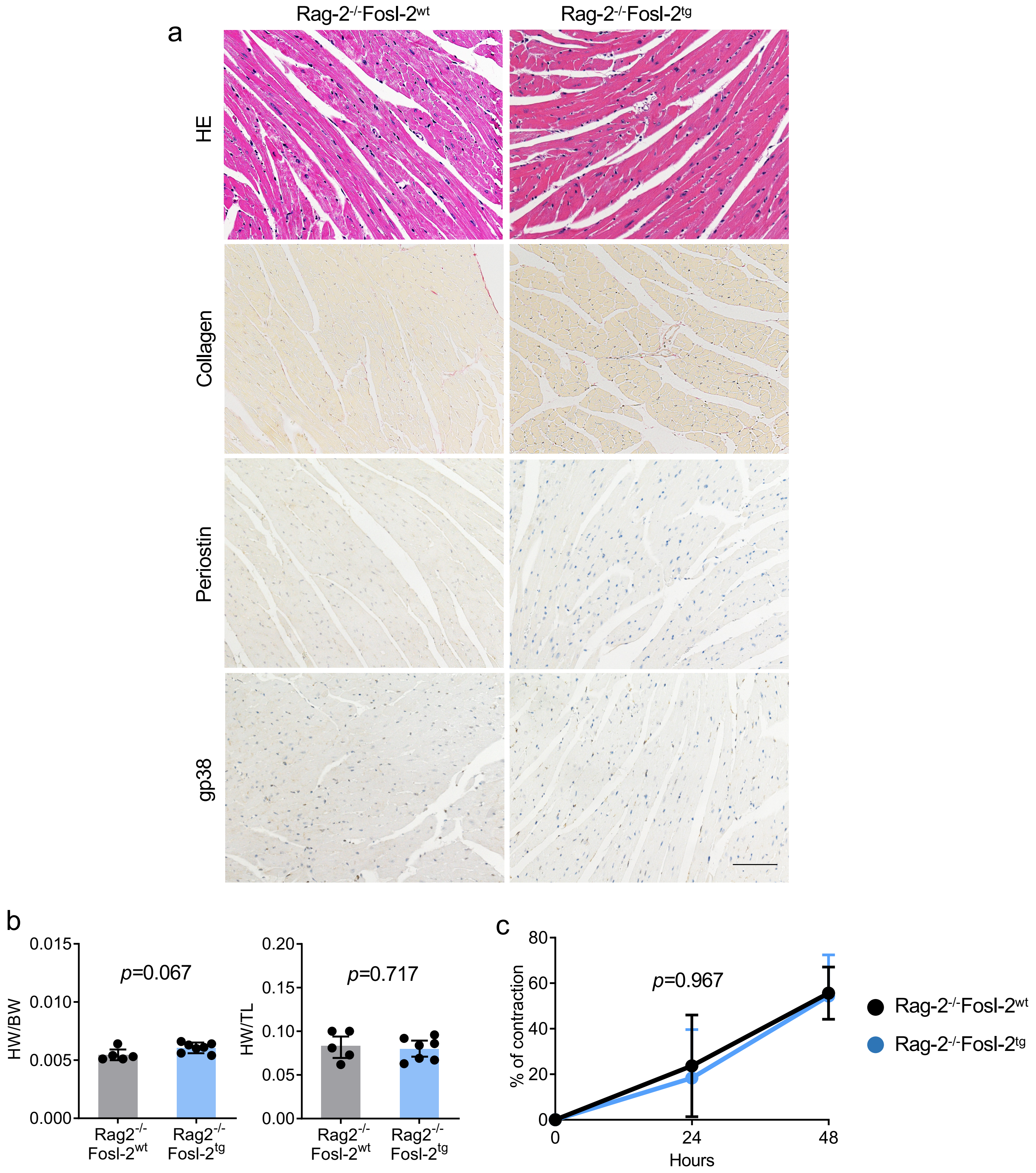

Supplementary Figure 11. Rag-2$^{-/-}$Fosl-2^{tg} mice displayed no fibrotic changes in the myocardium (a) Representative pictures of paraffin-embedded myocardial sections from 16-22-week-old mice stained for HE, Sirius Red for collagen, IHC for periostin and gp38 expression. Scale bar: 100 µm. Corresponding quantification is displayed in Figure 6C. (b) HW/BW (n=5-7, *Mann-Whitney test,* mean ± SEM) and HW/TL (n=5-7, *Mann-Whitney test,* mean ± SEM) ratios in Rag-2$^{-/-}$Fosl-2^{wt} and Rag-2$^{-/-}$Fosl-2^{tg} mice. (c) Contraction capacity of Lin^{-}gp38^{+}Rag-2$^{-/-}$Fosl-2^{wt} and Lin^{-}gp38^{+}Rag-2$^{-/-}$Fosl-2^{tg} fibroblasts (n=3, *two-way ANOVA* test with *Sidak's multiple comparisons test,* mean ± SEM).

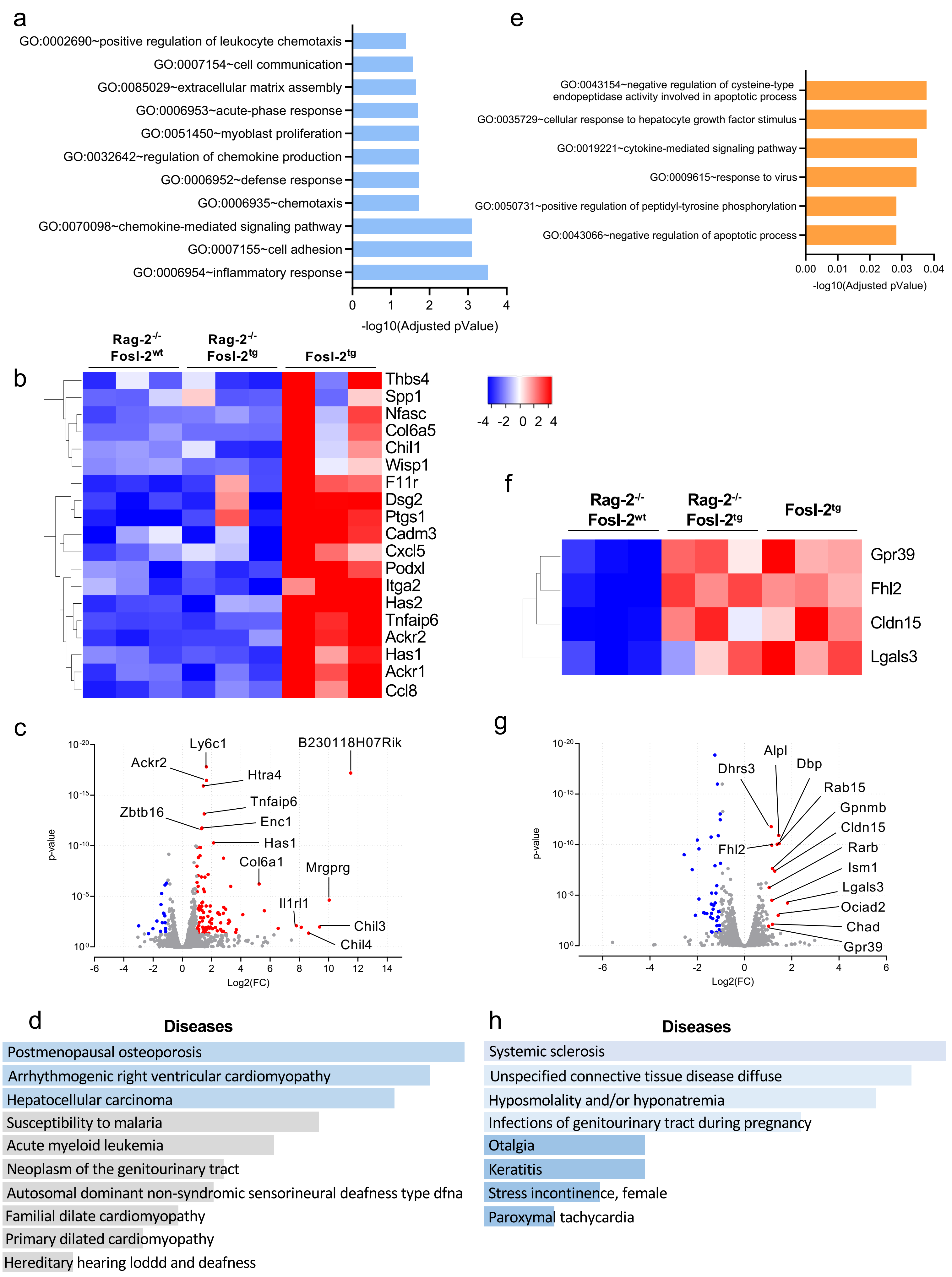
a
GO:0002690~positive regulation of leukocyte chemotaxis
GO:0007154~cell communication
GO:0085029~extracellular matrix assembly
GO:0006953~acute-phase response
GO:0051450~myoblast proliferation
GO:0032642~regulation of chemokine production
GO:0006952~defense response
GO:0006935~chemotaxis
GO:0070098~chemokine-mediated signaling pathway
GO:0007155~cell adhesion
GO:0006954~inflammatory response
0 1 2 3 4
-log10(Adjusted pValue)
e
GO:0043154~negative regulation of cysteine-type endopeptidase activity involved in apoptotic process
GO:0035729~cellular response to hepatocyte growth factor stimulus
GO:0019221~cytokine-mediated signaling pathway
GO:0009615~response to virus
GO:0050731~positive regulation of peptidyl-tyrosine phosphorylation
GO:0043066~negative regulation of apoptotic process
0.00 0.01 0.02 0.03 0.04
-log10(Adjusted pValue)
b
Rag-2-/- Fosl-2wt
Rag-2-/- Fosl-2tg
Fosl-2tg
Thbs4
Spp1
Nfasc
Col6a5
Chil1
Wisp1
F11r
Dsg2
Ptgs1
Cadm3
Cxcl5
Podxl
Itga2
Has2
Tnfaip6
Ackr2
Has1
Ackr1
Ccl8
-4 -2 0 2 4
f
Rag-2-/- Fosl-2wt
Rag-2-/- Fosl-2tg
Fosl-2tg
Gpr39
Fhl2
Cldn15
Lgals3
c
p-value
Ly6c1
B230118H07Rik
Ackr2
Htra4
Tnfaip6
Zbtb16
Enc1
Has1
Col6a1
Mrgprg
Il1rl1
Chil3
Chil4
Log2(FC)
g
p-value
Alpl
Dbp
Dhrs3
Rab15
Gpnmb
Cldn15
Fhl2
Rarb
Ism1
Lgals3
Ociad2
Chad
Gpr39
Log2(FC)
d
Diseases
Postmenopausal osteoporosis
Arrhythmogenic right ventricular cardiomyopathy
Hepatocellular carcinoma
Susceptibility to malaria
Acute myeloid leukemia
Neoplasm of the genitourinary tract
Autosomal dominant non-syndromic sensorineural deafness type dfna
Familial dilate cardiomyopathy
Primary dilated cardiomyopathy
Hereditary hearing loddd and deafness
h
Diseases
Systemic sclerosis
Unspecified connective tissue disease diffuse
Hyposmolality and/or hyponatremia
Infections of genitourinary tract during pregnancy
Otalgia
Keratitis
Stress incontinence, female
Paroxymal tachycardia

Supplementary Figure 12. Analysis of the transcriptome in Lin^{-}gp38^{+}Rag-2$^{-/-}$Fosl-2^{wt}, Lin^{-}gp38^{+}Rag-2$^{-/-}$Fosl-2^{tg} and Lin^{-}gp38^{+}Fosl-2^{tg} cardiac fibroblasts

(a) Gene ontology analysis of significantly deregulated genes in the comparison between Lin^{-}gp38^{+}Rag-2$^{-/-}$Fosl-2^{tg} vs. Lin^{-}gp38^{+}Fosl-2^{tg} fibroblasts. Cardiac fibroblasts were isolated for the hearts of 16-20-week-old mice. (b) Heatmap of selected significantly deregulated genes between Lin^{-}gp38^{+}Rag2$^{-/-}$Fosl-2^{tg} and Lin^{-}gp38^{+}Fosl-2^{tg} expressed in Transcripts Per Million (TPM) for Rag2$^{-/-}$Fosl-2^{wt}, Rag2$^{-/-}$Fosl-2^{tg} and Fosl-2^{tg} groups. The reported genes were involved in GO pathways: *Inflammatory response* and *Cell adhesion*. (c) Volcano plots of all significantly deregulated genes in the comparison between Lin^{-}gp38^{+}Rag-2$^{-/-}$Fosl-2^{tg} vs. Lin^{-}gp38^{+}Fosl-2^{tg} fibroblasts. (d) Analysis were performed with the comprehensive gene set enrichment web server EnrichR for *Diseases* (database ClinVar 2019) areas of significantly deregulated genes uniquely upregulated in Lin^{-}gp38^{+}Fosl-2tg vs. Lin^{-}gp38^{+}Rag2$^{-/-}$Fosl-2^{tg} fibroblasts. (e) Gene ontology analysis of significantly de-regulated genes in the comparison between Lin^{-}gp38^{+}Rag-2$^{-/-}$Fosl-2^{wt} vs. Lin^{-}gp38^{+}Rag-2$^{-/-}$Fosl-2^{tg} fibroblasts. (f) Heatmap of selected significantly deregulated genes between Lin^{-}gp38^{+}Rag-2$^{-/-}$Fosl-2^{wt} vs. Lin^{-}gp38^{+}Rag-2$^{-/-}$Fosl-2^{tg} fibroblasts, expressed in TPM for Rag2$^{-/-}$Fosl-2^{wt}, Rag2$^{-/-}$Fosl-2^{tg} and Fosl-2^{tg} groups. (g) Volcano plots of all significant significantly deregulated genes in the comparison between Lin^{-}gp38^{+}Rag-2$^{-/-}$Fosl-2^{wt} vs. Lin^{-}gp38^{+}Rag-2$^{-/-}$Fosl-2^{tg} fibroblasts. (h) Analysis were performed with the comprehensive gene set enrichment web server EnrichR for *Diseases* (database ClinVar 2019) areas of significantly de-regulated genes uniquely upregulated Lin^{-}gp38^{+}Rag2$^{-/-}$Fosl-2^{tg} vs. Lin^{-}gp38^{+}Rag2$^{-/-}$Fosl-2^{wt} .

a

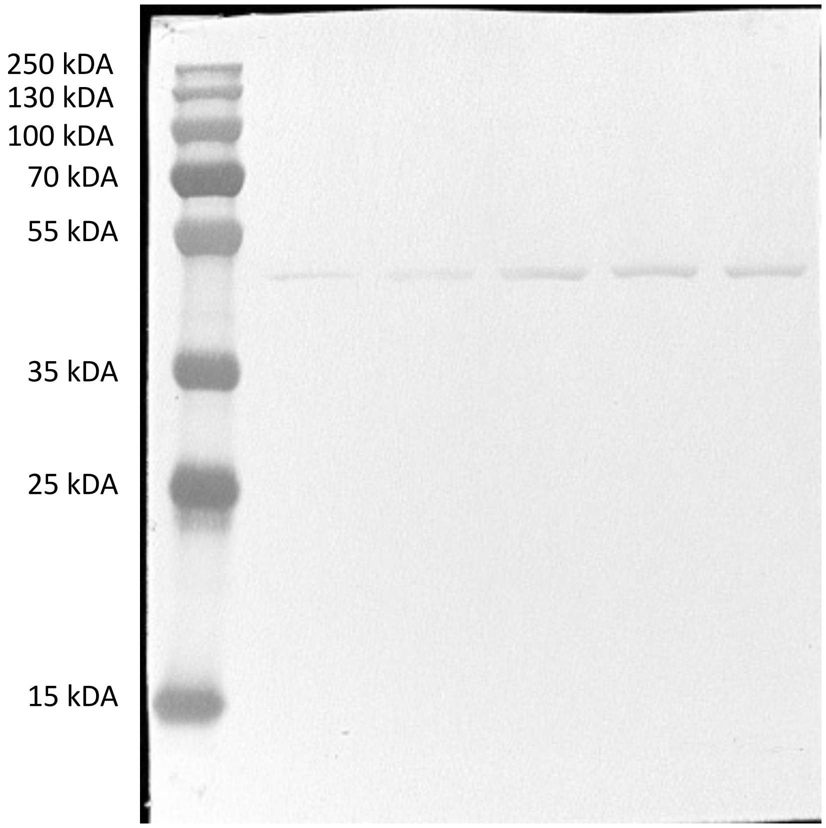

Supplementary Figure 13a. Representative Blot Marker for all Western blot pictures.

b

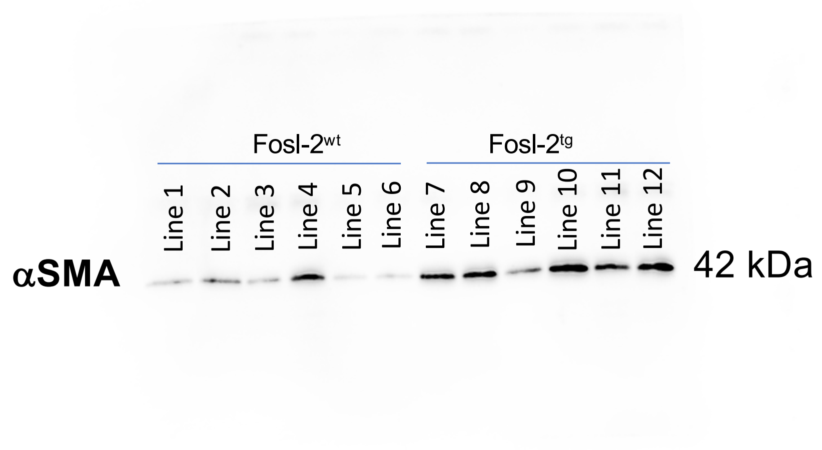

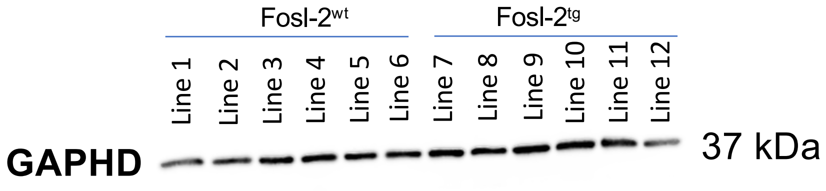

Supplementary Figure 13b. Original Western blot pictures for Figure 1b.

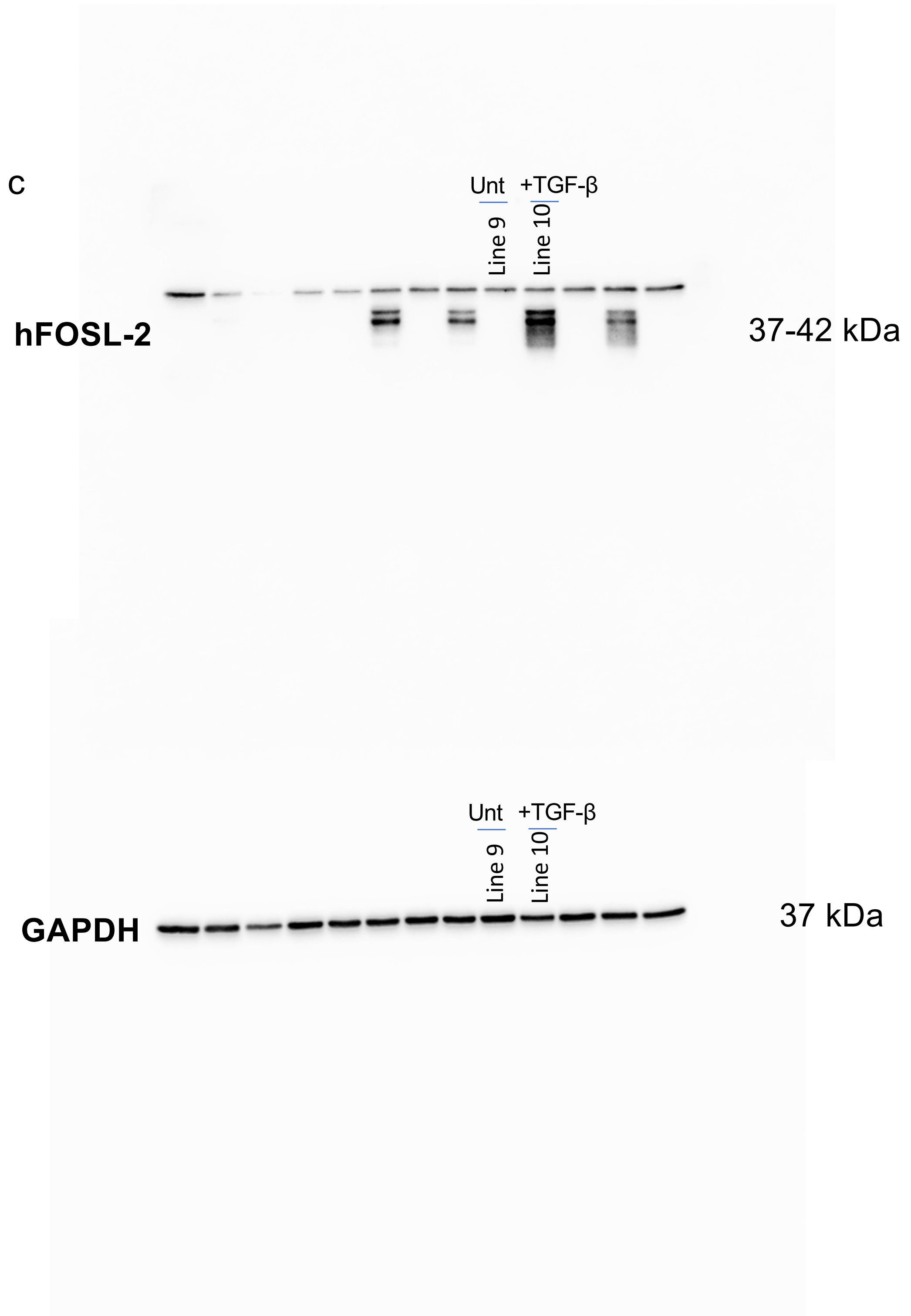

Supplementary Figure 13c. Original Western blot pictures for Figure 7e.

d

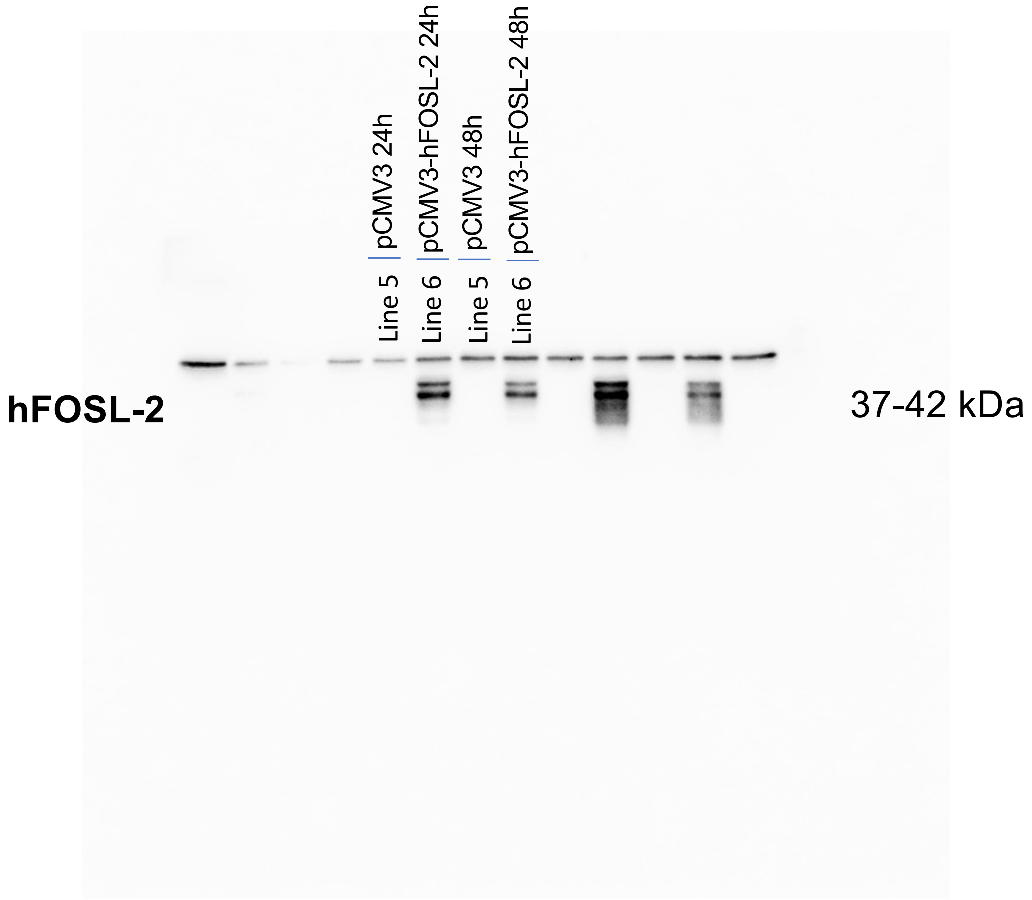

Supplementary Figure 13d. Original Western blot picture for Figure 7f.

e

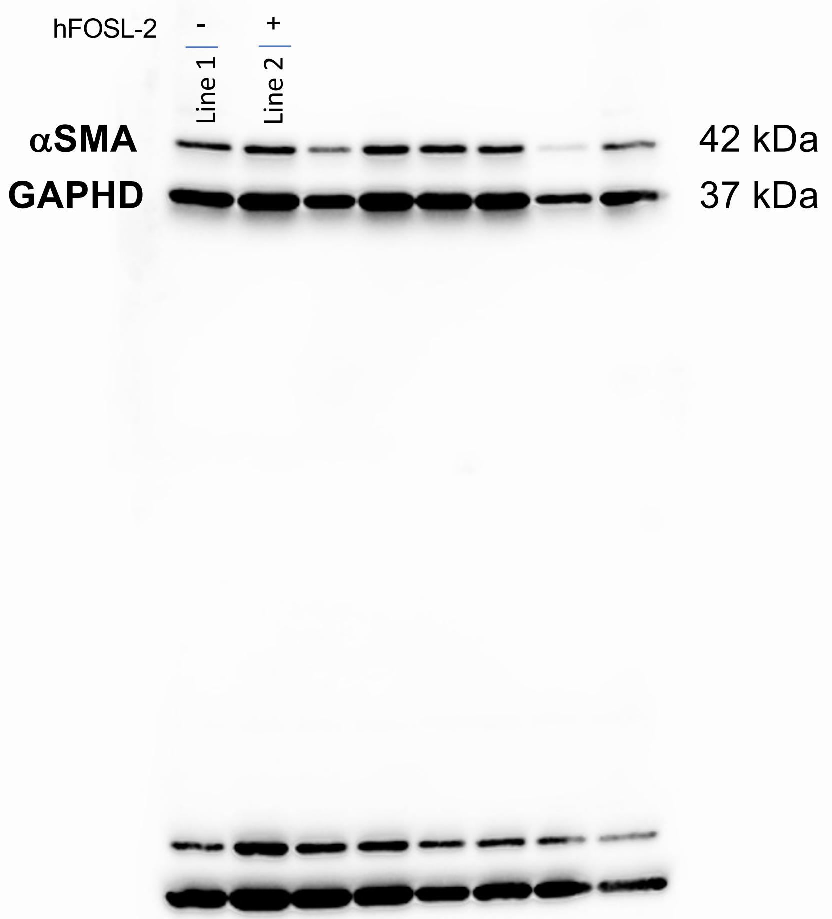

Supplementary Figure 13e. Original Western blot pictures for Figure 7g.

f

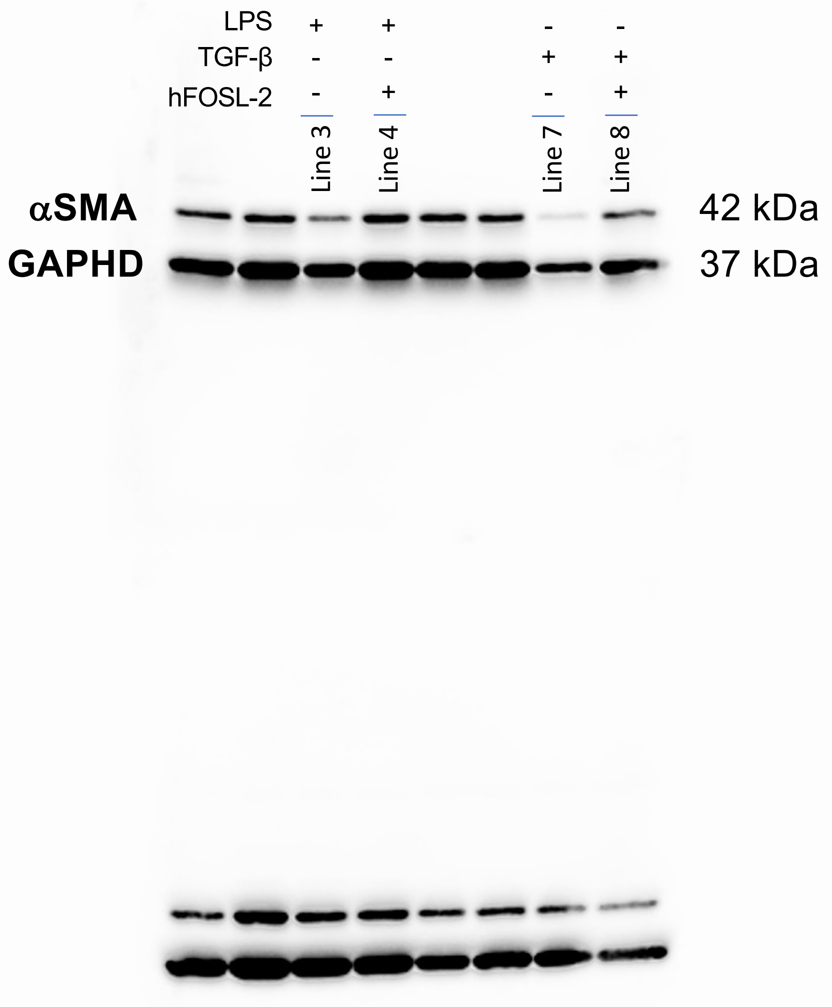

Supplementary Figure 13f. Original Western blot pictures for Figure 7h.

g

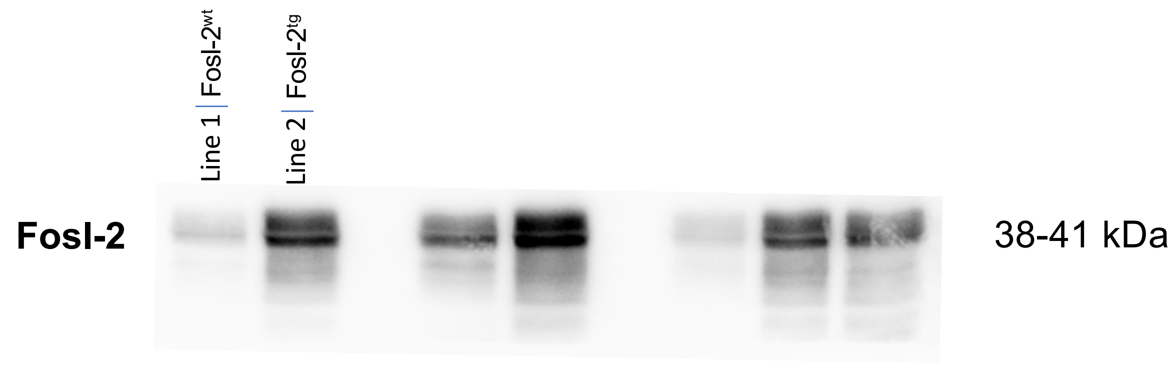

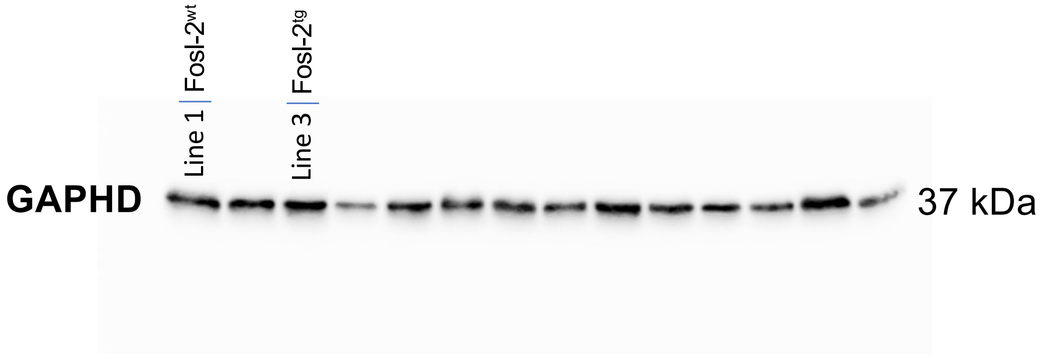

Supplementary Figure 13g. Original Western blot pictures for Supplementary Figure 1b.

h

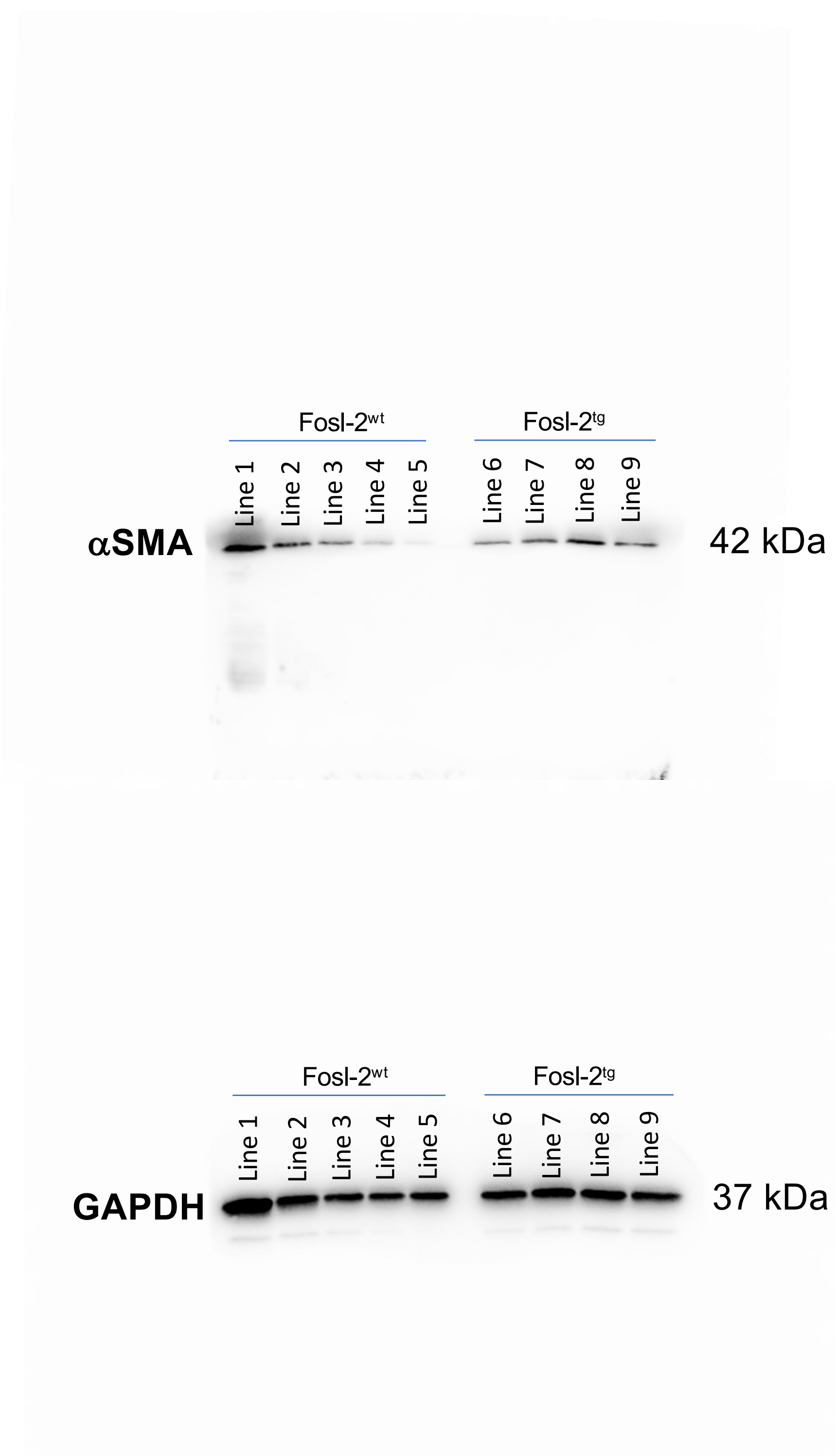

Supplementary Figure 13h. Original Western blot pictures for Supplementary Figure 3c.

i

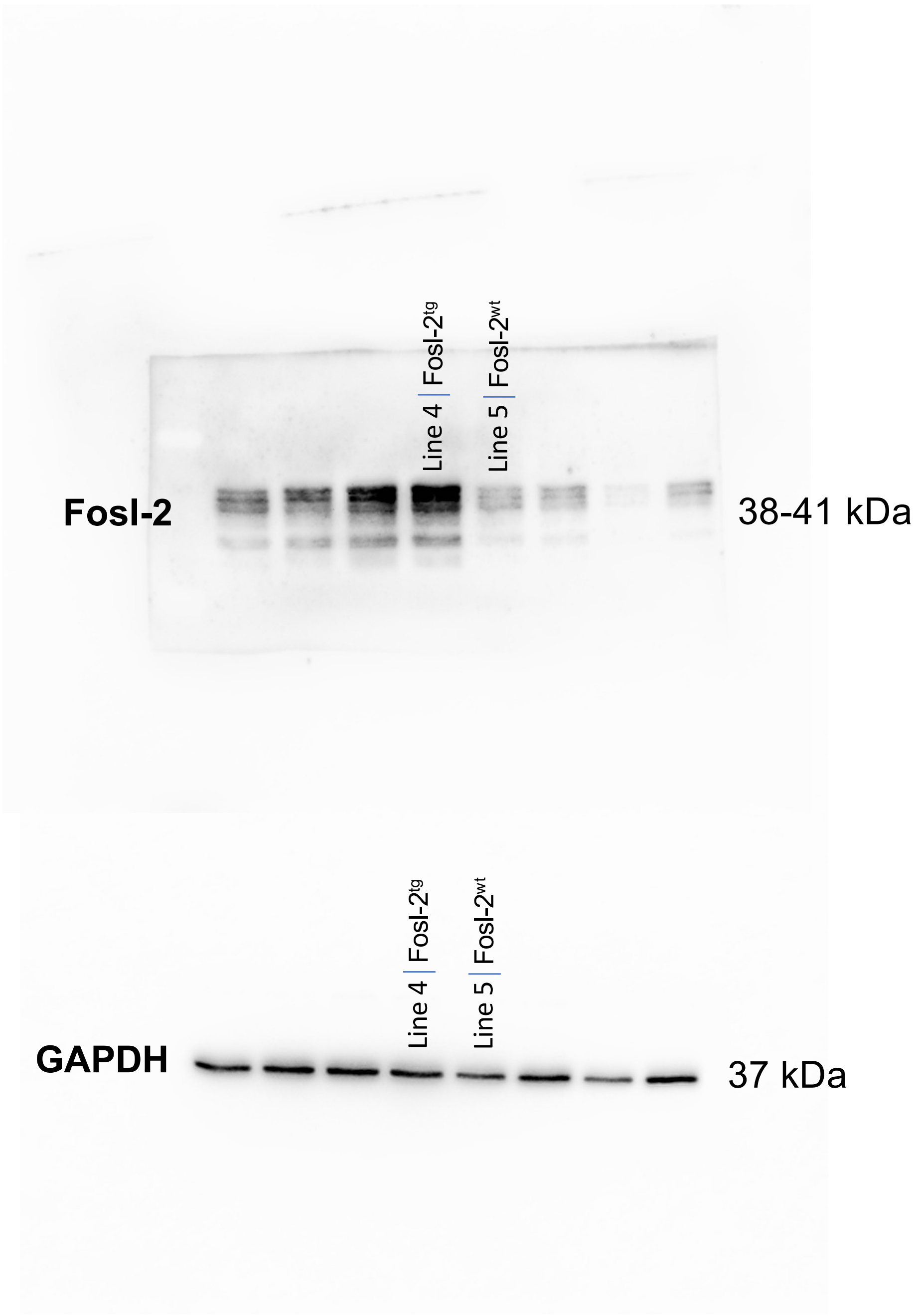

Supplementary Figure 13i. Original Western blot pictures for Supplementary Figure 4c.

j

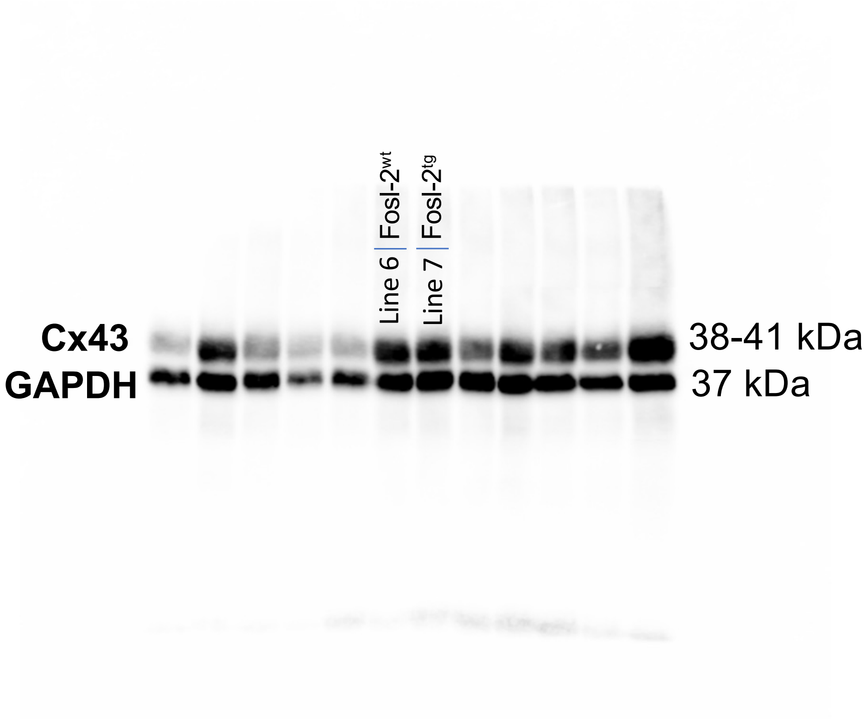

Supplementary Figure 13j. Original Western blot pictures for Supplementary Figure 9.

Supplementary Material and Methods

Supplementary Table 1. Characteristic of SSc patients

	All (n=24)	lcSSc (n=6)	dcSSc (n=18)
**Age** (mean±SD years)	43.3±10.9	47.7±9.1	41.8±11.2
**Sex**			
**Female** (n/N, %)	8/24 (33.3)	3/8 (37.5)	5/8 (62.5)
**Male** (n/N, %)	16/24 (66.7)	3/16 (18.75)	13/16 (81.25)
**Disease duration (mean±SD years)**	4.0±4.5	2.8±3.6	4.4±4.8
**ACR/EULAR 2013 criteria fulfilled** (n/N, %)	24/24 (100)	6/6 (100)	18/18 (100)
**mRSS at baseline visit** (mean±SD)	16.4±8.9	11.7±5.8	18.0±9.5
**Reason for the biopsy** (n/N, %)			
HSCT preparation	14/24 (58.3)	3/6 (50.0)	4/18 (22.2)
New PAH	1/24 (4.2)	0/6 (0)	1/18 (5.6)
New Arrhythmia	2/24 (8.3)	1/6 (16.7)	1/18 (5.6)
Cardiomyopathy	3/24 (12.5)	0/6 (0)	3/18 (16.7)
Tachycardia	1/24 (4.2)	1/6 (16.7)	0/18 (0)
**Organ involvement**			
**Lung**			
Pulmonary arterial hypertension by RHC (mPAP>20mmHg and PWP<15 mmHg) (n/N, %)	7/24 (29.2)	2/6 (33.3)	5/18 (27.8)
Lung fibrosis on HRCT (n/N, %)	21/24 (87.8)	3/6 (50.0)	18/18 (100)
**Cardiovascular system**			
MRI with pathology (yes, no) (n/N, %)	5/24 (20.8)	0/6 (0)	5/18 (27.8)
Arrhythmias (Triplets, VTs or any other higher grade arrythmia) in 24h ECG (n/N, %)	10/24 (41.7)	2/6 (33.3)	8/18 (44.5)
**Laboratory parameters**			
ANA (n/N, %)	24/24 (100)	6/6 (100)	18/18 (100)
Anti-centromere (n/N, %)	3/24 (12.5)	1/6 (16.7)	2/18 (11.1)
Anti-Scl-70 (n/N, %)	14/24 (58.3)	1/6 (16.7)	13/18 (72.2)
Anti-RNA-polymerase III (n/N, %)	1/24 (4.2)	0/6 (0)	1/18 (5.6)
Troponin positivity (yes, no) (n/N, %)	13/24 (54.2)	1/6 (16.7)	12/18 (66.7)
BNP elevation (yes, no) (n/N, %)	12/24 (50.0)	2/6 (33.3)	10/18 (55.6)
CK elevation (yes, no) (n/N, %)	13/24 (54.2)	3/6 (50.0)	10/18 (55.6)

Abbreviations:
HSCT: Hematopoietic stem cell transplantation
BNP: B-Typ Natriuretisches Peptid, Brain Natriuretic Peptide

Supplementary Table 2. Characteristic of control donors with healed myocarditis

	All (n=10)
**Age All** (mean±SD years)<br>**Female** (mean±SD years)<br>**Male** (mean±SD years)	50.6±11.6<br>49.5±12.9<br>55.0±1.4
**Sex**	
**Female** (n/N, %)<br>**Male** (n/N, %)	2/10 (20.0)<br>8/10 (80.0)
**Reason for the biopsy** (n/N, %)<br>Myocarditis?<br><br>**Biopsy result:** Healed myocarditis	10/10 (100)
**Interstitial myocardial fibrosis** (n/N, %)	0/10 (0)
**Active or chronic myocarditis** (n/N, %)	0/10 (0)
**EF <50%** (n/N, %)	2/10 (20.0)
**EF >50%** (n/N, %)	8/10 (80.0)

Supplementary Table 3. Cardiac function assessed by echocardiography

Echocardiography analyses of cardiac function of Fosl-2^{wt} and Fosl-2^{tg} mice at week 20-22. Abbreviations: bpm: beat per minute; LV vol, d: left ventricular end diastolic volume; LV vol, s: left ventricular end systolic volume; EF: ejection fraction; SV: stroke volume; CO: cardiac output; LVID, d: left ventricular end diastolic internal diameter; LVID, s: left ventricular end systolic internal diameter; FS: fractional shortening; MV E/A: peak Doppler blood inflow velocity across the mitral valve during early diastole (E) to peak Doppler blood inflow velocity across the mitral valve during late diastole (A) ratio. Mitral annulus measurements of motion velocity were used to assess E′, A′ ratios. *p* value was calculated with unpaired Student's *t-test.*

**Parameter**	**Fosl-2^{wt} (n of mice)**	**Fosl-2^{tg} (n of mice)**	*p* value
	Mean ± SD	Mean ± SD	(* *p<0.05*)
Heart Rate [BPM]	532 ± 54.5 (18)	444 ± 121 (22)	0.0039*
LV Area; s [mm^2]	2.53 ± 0.4 (18)	2.28 ± 0.6 (22)	0.120
LV Area; d [mm^2]	3.40 ± 0.4 (18)	3.78 ± 0.5 (22)	0.123
LV volume; s [µl]	23.8 ± 8.6 (18)	19.9 ± 11.9 (22)	0.255
LV volume; d [µl]	70.7 ± 15.8 (18)	60.0 ± 17.1 (22)	0.048*
Stroke Volume [µl]	46.9 ± 9.4 (18)	42.8 ± 12.1 (22)	0.258
Ejection Fraction [%]	66.8 ± 6.7 (18)	69.55 ± 15.4 (22)	0.134
Fractional Shortening [%]	36.9 ± 5.3 (18)	40.1 ± 11.2 (22)	0.269
Cardiac Output [ml/min]	24.5 ± 7.4 (18)	19.4 ± 7.7 (22)	0.041*
LV Mass [mg]	127.0 ± 30.9 (8)	137.3 ± 26.8 (11)	0.441
MV E/A	1.49 ± 0.3 (18)	1.67 ± 0.3 (22)	0.090
MV IVS E/E'	31.1 ± 8.9 (8)	28.3 ± 10.7 (11)	0.56
MV IVS E'/A'	0.95 ± 0.2 (8)	1.22 ± 0.5 (11)	0.147
HW/BW	0.008 ± 0.006 (18)	0.008 ± 0.0007 (22)	0.121
HW/TL	0.128 ± 0.09 (18)	0.09 ± 0.01 (22)	0.143
Disease phenotype score	0 score (18)	1 score (6)<br>2 score (6)<br>3 score (10)	

Supplementary Table 4. Murine primes used for RT-qPCR analyses

Gene	Forward Primer (5’ -> 3’)	Revers Primer (5’ -> 3’)
Fosl-2	GACCTGCAGTGGATGGTACA	GGATTGGACATGGAGGTG AT
Acta2	CGCTGTCAGGAACCCTGAGA	CGAAGCCGGCCTTACAGA
Col1α1	GATGACGTGCAATGCAATGAA	CCCTCGACTCCTACATCTTCTGA
Gapdh	CTGCACCACCAACTGCTTAGC	GGCATGGACTGTGGTCATGAG
Meis1	AGAACTTGAAGTAGGAAGGGA	ATCGTGGAGGGGATGCCTAG
Tbx3	GCTTCCATCGTGGGGACATC	GGCCGTAGTGGTCGAAATCT
Nos1ap	GGACGATGGGCACGATCTAC	ATCTCAACCCTGCTGTTGGG
Cdkn1a	CCAATGTGCGCTTGGAGTGA	CTGAGCGGCCTGAAGATTCC
Hand1	CAAAAAGACGGATGGTGGTCG	GGTGCGCCCTTTAATCCTCT
Sox5	AAAGATGGAGGCATGTGCGA	GGTGTCTCGTGCAAGGGTTA
Nfia	ACCAAGCCTCCAACCACATC	CACTGACGAATGGGTTTGCG
Scn10a	ATGACAGAGCAGAAGAAG	CCTGGTCACAATGTCAAACAC

Supplementary Table 5. Human primes used for RT-qPCR analyses

Gene	Forward Primer (5’ -> 3’)	Revers Primer (5’ -> 3’)
FOSL-2	ACCACCGTGGGCCGC	[illegible]TGCTCATCTCTCCTCC
ACTA2	ACAGAGTATTTG CGCTCCG	CCGACCGAATGCAGAAGGA
COL1a1	CAGCCGCTTCACCTACAG C	TTTTGTATTCAATCACTGTCTT GCC
GAPDH	GGGAAGCTTGTCATCAAT GGA	TCTCGCTCCTGGAAGATGGT

Supplementary Table 6. Antibodies utilised

Target (fluorochrome)	Clone	Company	Application	Dilution
Ter119 (PE)	TER-119	eBioscience (12-5921-81)	FACS	1:600
CD45 (PE)	30-F11	eBioscience (12-0451-82)	FACS	1:300
CD31 (PE)	390	eBioscience (12-0311-81)	FACS	1:300
gp38 (APC)	8.1.1	BioLegend (127409)	FACS	1:100
Ly6C (BV510)	HK1.4	BioLegend (128033)	FACS	1:400
Ly6G (PerCP-eFluor 710)	1A8-Ly6g	eBioscience (46-9668-80)	FACS	1:400
CD3 (BV421)	17A2	Biolegend (00228)	FACS	1:200
CD11b (APC)	M1/70	Biolegend (101211)	FACS	1:600
MHCII (PE-Cy7)	M5/114.15.2	eBioscience (25-5321-80)	FACS	1:600
Col1α1		Abcam (ab34710)	IHC,<br>IF	1:1000<br>1:200
αSMA	1A4	Sigma (A2547)	WB, IF	1:100
αSMA		Abcam (ab5694)	IHC	1:1000
Cx43		Abcam (ab11370)	WB, IHC,<br>IF	1:400<br>1:200
E-cadherin		Abcam (ab9465)	IF	1:100
Sarcomeric α-Actinin		R&D System (AF6426)	IF	1:100
GAPDH	D4C6R	Cell Signalling (#2118S)	WB	1:5000
Fosl-2	REY146C	Merk Millipore (MABS1261)	WB, IHC	1:500
Periostin	EPR6989(N)(B)	Abcam (ab14041)	IHC<br>IF	1:2000<br>1:200
GJA5	83004	Biorbyt (orb400460)	IF	1:100
gp38	8.1.1	eBioscience (14-5381-82)	IHC,<br>IF	1:10000<br>1:400
CONTACTIN-2	LDSO0119071	R&D System (AF4439)	IHC, IF	1:100
RGS4	GR136178-5	Abcam (ab135725)	IF	1:100
