# Supplementary material for: The AP-1 transcription factor Fosl-2 drives cardiac fibrosis and arrhythmias under immunofibrotic conditions
Source: Commun Biol. 2023 Feb 9;6:161. doi: 10.1038/s42003-023-04534-6 (PMC9911788; doi:10.1038/s42003-023-04534-6)
Supplement: Supplementary file 3 — Description of Additional Supplementary Files [file 42003_2023_4534_MOESM3_ESM.pdf]

Description of Additional Supplementary Files

File name: Supplementary Movie 1
Description: Mouse 1839_Severe arrhythmia_01 (PSLAX, M-Mode).

File name: Supplementary Movie 2
Description: Mouse 1839_Severe arrhythmia_02 (PSLAX, PW Doppler of PA).
