# Supplementary material for: The AP-1 transcription factor Fosl-2 drives cardiac fibrosis and arrhythmias under immunofibrotic conditions
Source: Commun Biol. 2023 Feb 9;6:161. doi: 10.1038/s42003-023-04534-6 (PMC9911788; doi:10.1038/s42003-023-04534-6)
Supplement: Supplementary file 6 — Reporting Summary [file 42003_2023_4534_MOESM6_ESM.pdf]

nature portfolio

Corresponding author(s): Gabriela Kania

Last updated by author(s): Dec 5, 2022

Reporting Summary

Nature Portfolio wishes to improve the reproducibility of the work that we publish. This form provides structure for consistency and transparency in reporting. For further information on Nature Portfolio policies, see our Editorial Policies and the Editorial Policy Checklist.

Statistics

For all statistical analyses, confirm that the following items are present in the figure legend, table legend, main text, or Methods section.

n/a	Confirmed	
☐	☒	The exact sample size (*n*) for each experimental group/condition, given as a discrete number and unit of measurement
☐	☒	A statement on whether measurements were taken from distinct samples or whether the same sample was measured repeatedly
☐	☒	The statistical test(s) used AND whether they are one- or two-sided<br>*Only common tests should be described solely by name; describe more complex techniques in the Methods section.*
☐	☒	A description of all covariates tested
☐	☒	A description of any assumptions or corrections, such as tests of normality and adjustment for multiple comparisons
☐	☒	A full description of the statistical parameters including central tendency (e.g. means) or other basic estimates (e.g. regression coefficient) AND variation (e.g. standard deviation) or associated estimates of uncertainty (e.g. confidence intervals)
☐	☒	For null hypothesis testing, the test statistic (e.g. *F*, *t*, *r*) with confidence intervals, effect sizes, degrees of freedom and *P* value noted<br>*Give P values as exact values whenever suitable.*
☒	☐	For Bayesian analysis, information on the choice of priors and Markov chain Monte Carlo settings
☒	☐	For hierarchical and complex designs, identification of the appropriate level for tests and full reporting of outcomes
☐	☒	Estimates of effect sizes (e.g. Cohen's *d*, Pearson's *r*), indicating how they were calculated

Our web collection on statistics for biologists contains articles on many of the points above.

Software and code

Policy information about availability of computer code

Data collection	Office Software, Vevo2100 Software Version 1.6.
Data analysis	ImageJ 1.47t, Office Software, EnrichR, FlowJo v.10 software, EzCG analysis software from eMouseSpecifics, Vevo2100 Software Version 1.6., Ponemah Physiology Platform System.

For manuscripts utilizing custom algorithms or software that are central to the research but not yet described in published literature, software must be made available to editors and reviewers. We strongly encourage code deposition in a community repository (e.g. GitHub). See the Nature Portfolio guidelines for submitting code & software for further information.

Data

Policy information about availability of data

All manuscripts must include a data availability statement. This statement should provide the following information, where applicable:

- Accession codes, unique identifiers, or web links for publicly available datasets
- A description of any restrictions on data availability
- For clinical datasets or third party data, please ensure that the statement adheres to our policy

RNA sequencing data were submitted to the ArrayExpress repository under the accession code E-MTAB-12271.

Human research participants

Policy information about studies involving human research participants and Sex and Gender in Research.

Reporting on sex and gender	Human endomyocardial biopsies (hEMBs) from SSc patients and control hEMBs from the patients with healed myocarditis (all data in Tables 1.1 and 1.2) were provided by the Cardiopathology, Institute for Pathology and Neuropathology, University Hospital Tubingen, Germany. The experiments with re-usage of human material were approved by Swissethics (BASEC-Nr. 2019-00058) and were performed in conformity with the principles outlined in the Declaration of Helsinki.
Population characteristics	all data in Tables 1.1 and 1.2
Recruitment	no recruitment since we re-used these myocardial biopsy from the Cardiopathology, Institute for Pathology and Neuropathology, University Hospital Tubingen, Germany
Ethics oversight	Swissethics: BASEC-Nr. 2019-00058

Note that full information on the approval of the study protocol must also be provided in the manuscript.

Field-specific reporting

Please select the one below that is the best fit for your research. If you are not sure, read the appropriate sections before making your selection.

☒ Life sciences ☐ Behavioural & social sciences ☐ Ecological, evolutionary & environmental sciences

For a reference copy of the document with all sections, see nature.com/documents/nr-reporting-summary-flat.pdf

Life sciences study design

All studies must disclose on these points even when the disclosure is negative.

Sample size

The example of sample size calculation:
Minimal group size was calculated to reach biologically meaningful effect. In this model, we consider changes in heart function measured as fractional shortening (FS) >50% of the effect observed in the reference group 2 (Cre+ mice) as biologically relevant. It results in n=20 mice per group.

Input data for calculation of detectable changes in FS for angiotensin II-induced heart inflammation and fibrosis model:
group A (baseline Cre- mice): FS = 30
group B (reference group: Cre+ mice): mean = 20, SD = 4.2
mean A - mean B = 10 => meaningful effect mean > 5 (assumed SD = 4.2)

Inputs for computation of the effect size d (two-sided t test of two independent means model):
Mean 1 = 20
Mean 2 = 25
SD 1 = 4.2
SD 2 = 4.2

Effect size d = 1.19

Inputs for computation of the group size (two-sided t test of two independent means):
alpha = 0.05
power = 0.95
effect size d = 1.19
allocation ratio N2/N1 = 1

Calculated in G*Power software

For all mice crossed with Rfpflox/flox mice:

Minimal group size was calculated to reach biologically meaningful effect. In this model, we consider changes in heart weight (HW)/body weight (BW) >30% of the effect observed in the reference group 2 (Cre+ mice with minipumps with angiotensin II) as biologically relevant. It results in n=9 mice per group.

Input data for calculation of detectable changes in HW/BW for angiotensin II-induced heart inflammation and fibrosis model:
group A (Cre+ mice with minipumps with saline): mean = 4 (HW/BW ratio measured in mg/g)
group B (reference group: Cre+ mice with minipumps with angiotensin II): mean = 6 (skin thickness in mm), SD = 0.4
mean B - mean A = 2, => meaningful effect for HW/BW mean > 0.6 (assumed SD =0.4)

Inputs for computation of the effect size d (two-sided t test of two independent means model):
Mean 1 = 6

Mean 2 = 5.4
SD 1 = 0.4
SD 2 = 0.4

Effect size d = 1.5

Inputs for computation of the group size (two-sided t test of two independent means):
alpha = 0.05
power = 0.95
effect size d = 1.5
allocation ratio N2/N1 = 1

Calculated in G*Power software

Data exclusions	No data were excluded
Replication	All attempt at replication were succesful
Randomization	Allocation was random. Group allocation/randomization: Animals will be randomly allocated to experimental groups prior to performing experiments using randomizer.org. and distribution of relevant parameters such as age and body weight will be comparable between all groups.
Blinding	The results will be analysed in double blinded manner.

Reporting for specific materials, systems and methods

We require information from authors about some types of materials, experimental systems and methods used in many studies. Here, indicate whether each material, system or method listed is relevant to your study. If you are not sure if a list item applies to your research, read the appropriate section before selecting a response.

Materials & experimental systems

n/a	Involved in the study	
☐	☒	Antibodies
☐	☒	Eukaryotic cell lines
☒	☐	Palaeontology and archaeology
☐	☒	Animals and other organisms
☒	☐	Clinical data
☒	☐	Dual use research of concern

Methods

n/a	Involved in the study	
☒	☐	ChIP-seq
☐	☒	Flow cytometry
☒	☐	MRI-based neuroimaging

Antibodies

Antibodies used	It is described in the Supplementary Table 5. "Antibodies utilised"
Validation	Validation: primary antibody and IgG control as secondary antibody

Eukaryotic cell lines

Policy information about cell lines and Sex and Gender in Research

Cell line source(s)	Foetal human cardiac fibroblasts were purchased from Sigma (Cell Applications),
Authentication	Sigma (Cell Applications)- data sheet
Mycoplasma contamination	Mycoplasma Test ThermoFisher Scientific
Commonly misidentified lines (See ICLAC register)	n/a

Animals and other research organisms

Policy information about studies involving animals; ARRIVE guidelines recommended for reporting animal research, and Sex and Gender in Research

Laboratory animals	Mus musculus
Wild animals	n/a
Reporting on sex	It is reported in the material section
Field-collected samples	n/a
Ethics oversight	Cantonal Veterinary Office Zurich had approved all animal experiments (ZH28/2015, ZH007/2019).

Note that full information on the approval of the study protocol must also be provided in the manuscript.

Flow Cytometry

Plots

Confirm that:

☒ The axis labels state the marker and fluorochrome used (e.g. CD4-FITC).

☒ The axis scales are clearly visible. Include numbers along axes only for bottom left plot of group (a 'group' is an analysis of identical markers).

☒ All plots are contour plots with outliers or pseudocolor plots.

☒ A numerical value for number of cells or percentage (with statistics) is provided.

Methodology

Sample preparation	It is described in the Supplementary methods
Instrument	Cells were analysed with the BD LSR Fortessa FACS (BD Biosciences) and sorted with FACS Aria III 4L (BD Biosciences).
Software	FlowJo v.10 software
Cell population abundance	It is described in the Result part
Gating strategy	Shown in Supplementary Figure 10.

☒ Tick this box to confirm that a figure exemplifying the gating strategy is provided in the Supplementary Information.
